# Development of Burnout Syndrome in Non-university Teachers: Influence of Demand and Resource Variables

**DOI:** 10.3389/fpsyg.2021.644025

**Published:** 2021-03-09

**Authors:** Marta Llorca-Pellicer, Ana Soto-Rubio, Pedro R. Gil-Monte

**Affiliations:** ^1^Department of Social Psychology, Unidad de Investigación Psicosocial de la Conducta Organizacional (UNIPSICO), University of Valencia, Valencia, Spain; ^2^Personality, Assessment and Psychological Treatments Department, University of Valencia, Valencia, Spain

**Keywords:** burnout, job stress, psychosocial risks at work, emotional labor, teachers, feelings of guilt

## Abstract

Psychosocial risks at work are an important occupational problem since they can have an impact on workers' health, productivity, absenteeism, and company profits. Among their consequences, burnout stands out for its prevalence and associated consequences. This problem is particularly noteworthy in the case of teachers. The aim of the study was to analyze the influence of some psychosocial factors (demand and resource variables) and risks in burnout development, taking into consideration the levels of burnout according to the Spanish Burnout Inventory (SBI). This paper contributes to advancing knowledge on this issue by analyzing the influence of work characteristics and personal characteristics on the progress of burnout. The sample consisted of 8,235 non-university teachers (2,268 men 27.5% and 5,967 women 72.5%), aged 22 to 70 (*M* = 45.16, *SD* = 9.18). For this purpose, statistical modeling by logistic regression was used. The results of this study showed that No burnout level was positively related with resources variables and negatively with demand variables. In the Medium-High levels and the higher levels of burnout (i.e., Profile 1 and Profile 2), there is a positive relation with demand variables and a negative one with resource variables. In conclusion, demand variables cause an increase in the burnout levels, influencing positively the movements between the levels of No burnout to Medium-High levels of burnout and Medium-High levels to Profile 1. At the same time, resource variables had a negative influence on burnout. However, the results in the movement between Profile 1 and Profile 2 were not expected. The variable Imbalance had a negative relationship with the movement between Profile 1 to Profile 2, and Social support and Autonomy at work had a positive relationship with this movement. Therefore, when professionals feel higher levels of burnout, lack of imbalance together with social support and autonomy could contribute to increased feelings of guilt and risk of higher burnout.

## Introduction

In the last few years, working conditions have fundamentally changed, such as how production is organized, high levels of quantitative and qualitative demands, preference on multifunction workers, or instability in the working relationship (Mero, 2018). These changes and other factors have caused some consequences that have a negative effect on work demands, such as emotional work (Junne et al., 2018), which has been gaining importance in recent years (Bhave and Glomb, 2016; Maxwell and Riley, 2017). Besides this, the change in conditions has caused an increase in psychosocial risk exposure, and the frequency of these factors can cause sick leave associated with work accidents or professional sickness (Fornell et al., 2018).

The increment of the psychosocial risks causes an increase in work-related stress (Junne et al., 2018). At present, work-related stress is one of the main workplace disorders of health and the main barrier to companies' and/or countries' growth (European Agency for Safety Health at Work, 2016). Therefore, work-related stress has consequences for the employees, their families, organizations, and countries (European Agency for Safety Health at Work, 2016). The costs to business and society are estimated to be in the billions of euros (European Agency for Safety Health at Work Eurofound, 2014).

In addition, several studies showed that work-related stress reduces social interaction, causes difficulties concentrating at work, produces physiological pain and cardiovascular problems, and increases mental illness in the form of depression and anxiety (Nielsen et al., 2018). Moreover, work-related stress has been related to the increase of turnover and absenteeism in the workplace, the decrease of the performance at work, or it may lead to the increase of workplace accidents (Nielsen et al., 2018).

A work-related stress consequence is burnout syndrome (Maslach et al., 2001; Elshaer et al., 2018). Burnout was defined by Maslach et al. (2001) as a psychological syndrome in response to chronic interpersonal and emotional stressors on the job. It is characterized to affect employees of the service sector such as teachers, police, nurses, doctors, etc. (Sarisik et al., 2019). The World Health Organization, following the International Classification of Disease (ICD) 11, defines burnout as a result of chronic workplace stress that the employees cannot manage (World Health Organization, 2019). Burnout is associated with different consequences such as absenteeism (Maslach, 2017; Gusy et al., 2019), health problems (Maslach, 2017; Simionato et al., 2019), increment in the mistakes during the shift (Stehman et al., 2019; Bakker and Wang, 2020), the job performance of the employee (Bakker and Demerouti, 2017; Bakker and Wang, 2020), and depression (Gil-Monte, 2012; Nagy et al., 2018; Hatch et al., 2019).

The prevalence of burnout in education oscillates between 11 and 35.5%, depending on the country and the study considered (Gil-Monte et al., 2011; Ratto et al., 2015; Villaverde et al., 2019). In Spain, several studies showed that 48% of educational professionals have high levels of burnout and 52% of educational professionals have moderate levels of burnout (Ruiz-Calzado, 2016). Other studies showed lower levels than the aforementioned; Unda et al. (2020) showed in their study a prevalence of burnout of 17.6%. “Some studies have related these levels of burnout in Spain to emotional variables such as Emotional exhaustion (Betoret, 2009) and Emotional intelligence (Puertas-Molero et al., 2018). Also, self-efficacy and coping resources play an important role in the management of factors such as work overload, role ambiguity and conflict, pressures of the teacher's role, inadequate resources, poor working conditions, lack of professional recognition, low remuneration, lack of involvement in decision making, lack of effective communication, staff conflicts, and pupil misbehavior (Betoret, 2009).”

Given this prevalence in recent years, the study of burnout in the education sector has become increasingly important (Kim and Burić, 2019; McLean, D., et al., 2019; Schonfeld et al., 2019). Most research has pointed out the importance of burnout on teachers (Kaur and Singh, 2014; Yerdelen et al., 2016; Salmela-Aro et al., 2019; Schonfeld et al., 2019; Mäkikangas et al., 2020; Pyhältö et al., 2021), considering it as a risk for teachers (Cecho et al., 2019) that can affect negatively their well-being (physically and psychologically) (Mousavy, 2014), effective teaching (Yerdelen et al., 2016), the interaction with students (Travers, 2001), their motivation for the job (McLean, L., et al., 2019), absenteeism (Makhdoom et al., 2019), depression (Martínez-Monteagudo et al., 2019), insomnia (Gu et al., 2020), or a decrease in the capacity to give support to the students (Jennings and Greenberg, 2009).

Several studies have focused on the study of specific burnout profiles in teachers (Salmela-Aro et al., 2019; Mäkikangas et al., 2020; Pyhältö et al., 2021). The present study uses as a framework, specifically, the model of Karasek (1979) in regard to the conception of the influence of demands and resources on the consequences of psychosocial risks, such as burnout. Also, regarding the characterization of burnout, the present study focuses on the model of Maslach and Jackson (1986), in conjunction with the model proposed by Gil-Monte (2012). Maslach and Jackson (1986) explained that burnout is formed by 3 dimensions: emotional exhaustion, depersonalization, and reduced personal accomplishment. Gil-Monte (2005) added to this three-dimensional model a new dimension, the Feeling of guilt (Gil-Monte, 2012; Maslach and Leiter, 2016; Rabasa et al., 2016). This model understands burnout as an emotional and cognitive deterioration that causes negative attitudes and behaviors toward clients or users of the organization as a coping strategy (Gil-Monte, 2005). From this model, Gil-Monte developed the Spanish Burnout Inventory (SBI) (Gil-Monte, 2011, 2019a) to evaluate the levels of burnout by 4 dimensions: Enthusiasm toward the job, Psychosocial exhaustion, Indolence, and Feelings of guilt. Enthusiasm toward the job is defined as the subject's desire to archive goals at work because it is a source of personal pleasure. Psychological exhaustion is defined as the appearance of emotions and physical exhaustion at work because the employees need to deal with clients with problems daily. Indolence is the appearance of negative attitudes of indifference and cynicism toward the organization's clients. And guilt is an appearance of guilt caused by the negative attitudes that the employee has developed for the work, especially toward the clients (Figueiredo-Ferraz et al., 2013). The combination of these components allows the identification of two burnout profiles and levels of severity of burnout between professionals (Gil-Monte, 2005).

Profile 1 includes subjects who report higher levels of psychosocial exhaustion, cynical behaviors—i.e., indolence—and cognitive deterioration but not higher feelings of guilt. So, it describes employees who suffer from stress and discomfort at work. In this profile, the cynical attitudes—i.e., indolence—are used as a coping strategy, and it allows the employee to control the stress levels and their consequences. Profile 2 includes employees who present higher levels of psychosocial exhaustion, cynical behavior, and cognitive deterioration and the highest levels of feelings of guilt. These employees experience discomfort more seriously at work (Gil-Monte, 2012). Coping strategies based on cynical attitudes are not as effective as in Profile 1. In addition, cynical attitudes and indolence in the relationships at work as coping strategies are perceived as inadequate (Guidetti et al., 2018).

Alongside these burnout levels, it is possible to consider two other levels of burnout. On the one hand, there are people who do not suffer from burnout (people with high levels of enthusiasm toward the job and lowest levels of psychological exhaustion and indolence), which we will call No-Burnout. On the other hand, there are people who do not suffer burnout but are close to suffering it (workers with lower levels of enthusiasm toward the job and higher levels of psychological exhaustion, and low levels of indolence and guilt), which we will call Medium-High levels of burnout. Guidetti et al. (2018) found some profiles of burnout fitting these levels. The existence of these levels and profiles is key, given that each of the profiles requires different intervention patterns (Guidetti et al., 2018), as well as different levels of risk and different associated consequences for the worker, the company, and society as a whole (Jin et al., 2015; Abósa et al., 2019).

Despite their importance, Guidetti et al. (2018), in their study, did not analyze which variables predicted the appearance of one type of level of burnout or another, nor which variables predicted the change from one level to another. Therefore, the importance of burnout in teachers is clear, as well as the need to have levels that allow understanding and classification of the employees, focusing the coping strategies that the employees are using, thus focusing the resources that employees need to use depending on the case (Guidetti et al., 2018).

Although different studies highlight the importance that various factors can have in the appearance of burnout, no study analyzes which factors determine the greater probability of belonging to one level or another. Karasek model 1979 has the most theoretical and empirical support, and it is the one that currently has the most influence and attention. It explains work-related stress according to the imbalance between psychology demands at work (e.g., workload, interpersonal conflicts, imbalance, role ambiguity, and role conflict) and the control level or resources that the employee has (e.g., autonomy, feedback, etc.). Johnson and Hall (1988) added the variable social support as a third dimension of control. According to this model, the employee's health or well-being depends on the balance of the work demands and the personal resources that the employee has. When the demands are higher than the resources, it can cause a feeling of work-related stress by the employee. In addition, chronic work-related stress can cause burnout syndrome, which is able to appear as several disorders of health or psychosomatic symptoms.

The following demands stand out for their importance: interpersonal conflicts (Skaalvik and Skaalvik, 2007), workload (Alarcon, 2011), imbalance (Taris et al., 2001; Backhaus et al., 2018), role conflict, and role ambiguity (Alarcon, 2011). These demand variables are positively correlated with burnout (Backhaus et al., 2018; Engelbrecht et al., 2019; McCarty et al., 2019; Xu, 2019; Klein et al., 2020). Moreover, in recent years another demand has emerged amongst the others, emotional labor (Pisaniello et al., 2012; Yilmaz et al., 2015).

Skaalvik and Skaalvik (2007), in their study among teachers, showed that conflicts with parents and pupils were the conflicts that generate the most tension in teachers. The study found a negative relation between tension and self-efficacy and its manifestation in psychological exhaustion and depersonalization. On the other hand, Gonçalves et al. (2019) found a relationship between burnout and workload, and this variable was a predictor of emotional exhaustion. Unda (2010), in her study, found a negative relation with enthusiasm toward the job and positive relation with emotional exhaustion, guilt, and indolence. In addition, Xu (2019), in her study among education professionals, found a relation between role conflict and emotional exhaustion and depersonalization and added that role conflict would decrease the enthusiasm and energy for work. Also, Chung and Choo (2018), in their study, found a relationship between role ambiguity and emotional exhaustion, and it has a negative relation with personal accomplishment.

Finally, as previously stated, in recent years, emotional labor has increased its importance in the research (Pisaniello et al., 2012; Yilmaz et al., 2015). Several studies have shown the relationship between emotional labor and burnout (Pisaniello et al., 2012; Andela et al., 2015; Yilmaz et al., 2015). Yilmaz et al. (2015) that, in teachers, natural-felt emotions and surface acting predicted the burnout dimension, emotional exhaustion, and depersonalization. In addition, emotion suppression and emotion dissonance were related to burnout (Andela et al., 2015). Moreover, Pisaniello et al. (2012) found a positive relationship between surface acting and emotional exhaustion and depersonalization and a negative relation with personal accomplishment. Wegge et al. (2010), in their study, explained a positive relationship between emotional dissonance with emotional exhaustion and depersonalization and a negative relation with personal accomplishment.

Resource variables have been negatively related to burnout (Khan et al., 2018; Hatch et al., 2019). Resource variables highlighted are job autonomy, social support, and resources at work (Setti et al., 2016). Some variables, such as social support, are considered as a protective factor against burnout (Setti et al., 2016). Thus, an excess of demands will produce a negative consequence in the employee, as higher burnout, however having enough resources benefit the employee, decreasing the probability of having higher burnout (Hatch et al., 2019).

In addition, some sociodemographic variables can be related to burnout, such as sex (Lebares et al., 2018), age (LaFaver et al., 2018), and level of education (Langher et al., 2017; Smetackovaa, 2017). Sex is a variable widely studied in relation to burnout (Lebares et al., 2018). Different studies in teacher samples found that women have higher levels of burnout than men (Alavinia and Ahmadzadeh, 2012; Leineweber et al., 2013). In addition, a meta-analyses study showed that women are more likely to report burnout than men (Purvanova and Muros, 2010). Also, some studies showed that the variable age has a positive relation with burnout -i.e., with increasing age comes increasing burnout. However, it changes when the employees become middle-aged, which decreases the levels of burnout (LaFaver et al., 2018). Different levels of education showed despairing results. Some studies did not find any difference between education levels (Smetackovaa, 2017), and other studies showed a higher level of burnout in secondary school (Langher et al., 2017).

The importance of burnout syndrome has been widely proved, especially in teachers. However, no study has been observed that analyzes the role of demands, resources, and sex, emphasizing the emotional burden of work in the appearance of different burnout levels. Neither has there been a study that analyzes which factors are more important when considering the move from one burnout profile to another. For all these reasons, the present study is particularly interesting when trying to fill this gap by analyzing the role played by the demands of work, resources, sex, age, and levels of education in the appearance of each burnout profile, considering the levels of guilt, and at the same time analyzing what factors could predict the evolution from more harmful profiles to healthier ones on teachers.

**Figure 1:**
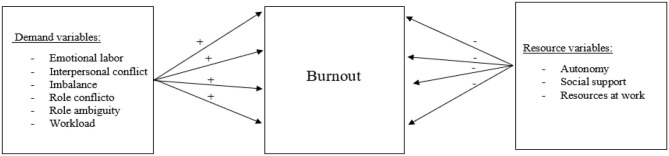
Hypothesis 1 and 2.

We hypothesized that:

Hypothesis 1: A significant positive relationship is expected between psychosocial demands at work and burnout, i.e., higher prevalence levels on demand variables will increase the probability to have higher levels of burnout (Figure 1).

Hypothesis 2: A significant negative relationship is expected between psychosocial resources at work and burnout, i.e., higher prevalence levels on resource variables will increase the probability to have lower levels of burnout (Figure 1).

**Figure 2:**
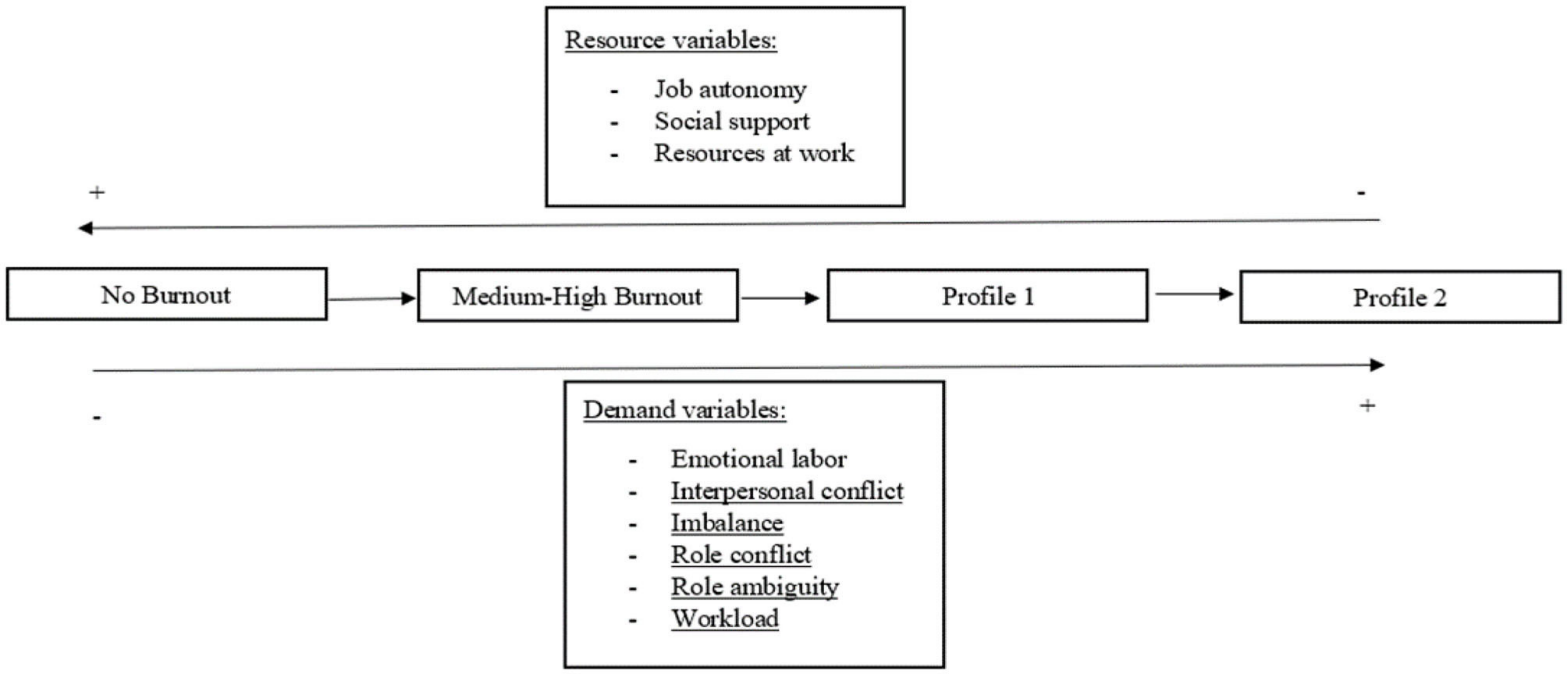
Hypothesis 3.

Hypothesis 3: Higher levels on psychosocial demand variables will predict the move toward higher levels of burnout (No burnout → Medium-High levels → Profile 1 → Profile 2). At the same time, psychosocial resource variables will be predictors of the moves for healthier burnout levels (Profile 2 → Profile 1 → Medium-High level → No burnout) (Figure 2).

## Materials and Methods

### Participant*s*

A total of 8,235 non-university teachers of different levels of public education from the Community of Valencia (Spain) participated in this study (72.5% female). Aged: *M*_*age*_ = 45.16 years, *SD* = 9.18, Range: 22–70 years and with a seniority: *M*_*seniority*_ = 17.41 years, *SD* = 10.33, Range: 0–45 years. The sample was distributed in 17.1% of kindergarten teachers, 39.9% primary school teachers, 43% secondary school teachers and trade school teachers, and 12.9% were other categories. The sample was selected by the Instituto Valenciano de Seguridad y Salud en el Trabajo (INVASSAT) (Valencian Health & Safety at the Workplace Institute). The inclusion criteria were: (a) to be working, not to be on sickness leave, (b) to belong to a school in the Valencian Community that is not a university, and (c) to accept to participate in the study.

In the cluster analyses, the total sample in each burnout level was: No burnout level, 3.536 teachers; Medium-High level, 3.887 teachers; Profile 1 level, 597 teachers; and Profile 2 level 215 teachers.

### Instruments

The UNIPSICO questionnaire (Gil-Monte, 2016a,b) evaluates: Role ambiguity (α = 0.79; ω = 0.75; CI: 0.741–0.764) by 4 items inverse (e.g., “*I know what my responsibilities are*”) (items were inversed to carry out the analyses to estimate Role ambigüity); Role conflict (α = 0.70; ω = 0.71; CI: 0.696–0.718) was composed of 5 items (e.g., “*I receive incompatible request from two or more people*”); Workload (α = 0.72; ω = 0.740; CI: 0.735–0.753) was evaluated by 6 items (e.g., “*Do you think you have to do a job that is too difficult for you?*”); Imbalance (α = 0.79; ω = 0.80; CI: 0.794–0.809) was evaluated by 5 items (e.g. “*I am rewarded very little for the effort I make at work*”); Interpersonal conflicts (α = 0.72; ω = 0.72; CI: 0.703–0.731) was composed of 6 items (e.g. “*How often do you have conflicts with your students*?”); Autonomy (α = 0.84; ω = 0.81; CI: 0.780–0.816) was evaluated by 5 items (e.g., “*I think my job gives me enough autonomy*”); Social support (α = 0.84; ω = 0.88; CI: 0.870–0.881) was composed of 6 items (e.g., “*How often do your supervisor helps you when problems arise at work?”*). Resources at work was evaluated by 7 items (e.g., To “*what extend in your workplace there are technological resources*”) (α = 0.83; ω = 0.80; CI: 0.796–0.810). The items were answered on a scale of 5 points of frequency ranging from 0 (Never) to 4 (Very frequently: every day). Emotional labor (12 items, α = 0.79; ω = 0.81; CI: 0.799–0.814), employees' ability to cope in an effective way with the stressful situation because they can use their own coping resources and skills, was evaluated by a short version of the Frankfurt Emotion Work Scale (FEWS) (Zapf et al., 1999) taken from the adapted 21 items Spanish version (Ortíz et al., 2012), that evaluated 6 dimensions: Positive emotions (2 items, e.g., “*Requirement to express pleasant emotions to the pupils and their families*”), Negative emotions (2 items, e.g., “*Requirement to express unpleasant emotions to the pupils and their familiars*”), Neutral emotion (2 items, e.g., “*Requirement to be neutral and impartial with the pupils*”), Sensitivity requirements (2 items, e.g., “*Requirement to be sensitive to the feelings of pupils*”), Interaction control (2 items, e.g., “*Duration of the interaction independent of the customer's feelings*”), and Emotional dissonance (2 items, e.g., “*Can you interrupt an interaction with a customer?*”).

Spanish Burnout Inventory (Gil-Monte, 2011; Figueiredo-Ferraz et al., 2013) has 20 items distributed in 4 dimensions; Enthusiasm toward the job, defined as the individual's desire to achieve goals at work as a source of personal pleasure (5 items; e.g., “*I find my work quite rewarding*”), this scale is similar to that of the Personal accomplishment of the MBI; Psychological exhaustion, defined as the appearance of emotional and physical exhaustion due to the necessity to deal with people or problems (4 items; e.g., “*I feel weighed down by my job*”), this scale is similar to that of the Emotional exhaustion of the MBI; and Indolence defined as the appearance of negative attitudes of indifference and cynicism toward the patients (6 items; “*I don't like taking care of some inmates”*), this scale is similar to that of the Depersonalization of the MBI. According to SBI Manual, global burnout is the mean of the 15 items from the subscales of Enthusiasm toward the job (reversed), Psychological exhaustion, and Indolence. This scale has correct reliability (α = 0.85; ω = 0.81; I CI: 805–0.823). In addition, Guilt was evaluated as a subscale of SBI. It is evaluated by 5 items (e.g., “*I regret some of my attitude at work*”) (α = 0.72; ω = 0.73; CI: 0.712–0.739). Low scores on Enthusiasm toward the job, along with high scores on Psychological exhaustion and Indolence, indicate high levels of burnout. Items are answered on a 5-point frequency scale, ranging from 0 to 4 (Never-Very frequently: Every day). Higher feelings of Guilt account for the difference between Profile 1 -i.e., high global burnout and low feelings of guilt- and Profile 2 -i.e., high global burnout and higher feelings of Guilt, values equal or higher than percentile 89th (see Data analysis section).

### Procedure

This study respected the fundamental principles of the Declaration of Helsinki (World Medical Association, 2013), with particular emphasis on the anonymization of the data collected, confidentiality, and non-discrimination of participants. All the educational non-university centers in the Community of Valencia were asked to participate, and the teacher's collaboration was voluntary. Teachers were informed about the purpose of the study, the possible benefits, and possible consequences of their participation.

This study was part of a psychosocial assessment of the Instituto Valenciano de Seguridad y Salud en el Trabajo (INVASSAT). In all cases, regional government instructions were followed. Previously to start the assessment, the ethical department of this institution was consulted and concluded that as the participation was voluntary and the teachers just needed to answer an anonymous questionnaire, the research did not need to be checked by a bioethics committee. The data was collected between October 2015 and March of 2018 by employees working in the INVASSAT. The INVASSAT employees went to each educational center and informed the director, union representative, and teachers of each school of the procedure, explaining the confidentiality, anonymity, and privacy of the study. The INVASSAT employees convened a meeting with all the teachers of each school and explained the questionnaire. Then each teacher did the questionnaire individually. The questionnaire was done in the presence of the INVASSAT employees to answer any questions the teachers could have. When the teachers finish the questionnaire, they gave them to the INVASSAT employee. The time of completion of the questionnaire was around an hour and a half.

### Data Analysis

The present research is a correlational study. In order to achieve the objectives proposed in the study, the participants were first classified into 4 levels of burnout: No burnout, Medium-High levels of burnout, Profile 1 and Profile 2 of burnout. According to the SBI Manual (Gil-Monte, 2019a), there are 6 levels of burnout: (1) Very low level of burnout (values equal to or lower than percentile 10th), (2) Low levels of burnout (between percentile 10th to values equal to or lower than percentile 33rd), (3) Medium levels of burnout (between values of percentile 33rd to equal to or lower than percentile 66th), (4) High levels of burnout (values higher than percentile 66th to equal or lower than percentile 89th). These levels are the result of the mean of the 15 items of the SBI inventory's subscales Enthusiasm toward the job (reversed; 5 items), Indolence (6 items), and Psychological exhaustion (4 items) (5). Profile 1 (values higher than percentile 89th' i.e., critical levels of burnout, and Guilt values equal or lower than percentile 89th) and (6) Profile 2 (values higher than percentile 89th and Guilt values higher than percentile 89th, i.e., critical levels of burnout and critical levels of Guilt) (Gil-Monte, 2019a; Misiolek-Marín et al., 2020). In addition, to calculate Profile 1 and 2, it is being used to differentiate between both profiles the levels of Guilt. Thus, the clusters in our study were established according to the Manual instruction in:

- No burnout level: It was formed by Very low and Low levels of global burnout (in this level, teachers were included with percentiles between 0th and 33rd).
- Medium-High levels: It was formed by Medium and High levels of burnout (in this level, teachers were included with percentiles between 33rd and 89th).
- Profile 1: It was formed by critical levels of burnout but no critical levels of guilt (in this level, there were teachers with burnout percentiles higher than percentile 89th, and Guilt percentiles were equal or lower than percentile 89th).
- Profile 2: It was formed by critical levels of burnout and guilt (in this level, there were teachers with percentile higher than percentile 89th with guilt values higher than percentile 89th).

After distributing the subjects in the 4 levels of burnout, we proceeded to analyze the influence of the Sex, the Age and Level of education, resource variables, and demand variables on levels of burnout and to ascertain what factors explain the likelihood of a participant to be in 1 level rather than another. Firstly, ANOVA with Turkey *post-hoc* was made to compare differences in the demand and resources variables according to the different levels. Secondly, a logistic regression with three steps was carried out for the prediction of 4 levels of burnout. In the first step, the variables Sex, Age, and Level of education were introduced, then in the second step, demand variables were introduced (Emotional labor, Interpersonal conflict, Imbalance, Role conflict, Role ambiguity, and Workload). Finally, in the third step, the resource variables were introduced (Autonomy, Social support, and Resources at work). Thirdly, logistic regression was done to find the relevant factors that explain the likelihood of a participant to be in one level rather than another. Performing logistic regression by grouping and comparing the groups allows differences between such groups to be identified, and thus to know the difference that would explain the change from a group to another. Specifically, the change from No burnout level (percentile 0th to percentile 33rd) to Medium-High level (higher than percentile 33rd to percentile 89th); Medium-High level to Profile 1 level (higher percentile 89th with guilt values equal or lower than percentile 89th) and Profile 1 level to Profile 2 level (higher than percentile 89th with guilt values higher than percentile 89th) was compared. The variables Sex, Age, and Level of education were used to control the effect they had on the other variables.

## Results

First, to observe the differences in demands and resources depending on the levels of burnout, an ANOVA analysis with HDS Tukey *post-hoc* was performed. These results (Table 1) showed differences (*p* < 0.001). No study has been made that analyzes the role of demands with UNIPSICO dimensions according to the different levels of burnout. In reference to the F effect size ($\eta_{\text{p}}^{2}$), each variable had a moderate effect (≥0.06) (Cárdenas and Arancibia, 2014), except the variables: Interpersonal conflicts, Role conflicts and Workload that could be considered big effect (≥0.14) (Cárdenas and Arancibia, 2014) and Emotional labor that could be considered small effect (≤0.01). In the *post-hoc* analyses, all the variables showed a significant effect (*p* < 0.05), except the Resources variables (Autonomy, Social support, and Resources at work) and the variable Emotional labor and Role conflict between variables Profile 1 and Profile 2 (*p* > 0.05). Therefore, the Resources variables did not have a significant influence on those levels of burnout. In *post-hoc* results, the Demand variables, with the exception of Emotional labor and Role conflict, showed a difference between the mean in all the levels of burnout (Table 1).

**Table 1:** ANOVA between profiles and predictor.

	**No burnout**		**Medium-High levels**		**Profile 1**		**Profile 2**		**F_**(3, 8, 231)**_**	**$\eta_{\text{p}}^{2}$**	**Power**	**1 vs. 2**	**1 vs. 3**	**1 vs. 4**	**2 vs. 3**	**2 vs. 4**	**3 vs. 4**
	**M**	**SD**	**M**	**SD**	**M**	**SD**	**M**	**SD**									
EL	2.49	0.61	2.56	0.58	2.69	0.63	2.77	0.54	32.42***	0.01	1	***	***	***	***	***	
IC	0.45	0.43	2.83	0.46	0.98	0.56	1.24	0.63	462.20***	0.14	1	***	***	***	***	***	***
Imbalance	1.60	0.67	1.91	0.61	2.31	0.65	2.17	0.65	296.68***	0.10	1	***	***	***	***	***	*
RC	0.84	0.61	1.20	0.67	1.75	0.83	1.88	0.75	499.50***	0.15	1	***	***	***	***	***	
RA	3.48	0.55	3.15	0.64	2.79	0.78	2.61	0.82	382.21***	0.12	1	***	***	***	***	***	***
Workload	1.54	0.50	1.83	0.48	2.16	0.57	2.29	0.51	461.98***	0.14	1	***	***	***	***	***	**
SS	3.19	0.77	2.85	0.82	2.33	0.92	2.46	0.83	269.07***	0.09	1	***	***	***	***	***	
Autonomy	2.93	0.48	2.68	0.54	2.34	0.64	2.42	0.57	313.22***	0.10	1	***	***	***	***	***	
Resources	2.48	0.69	2.22	0.67	1.84	0.73	1.92	0.73	204.25***	0.07	1	***	***	***	***	***	

EL, Emotional labor; IC, Interpersonal conflict; WO, Workload; SS, Social support; RC, Role conflict; RA, Role ambiguity;

* p ≤ 0.05,

** p ≤ 0.01,

*** p ≤ 0.001.

*1 vs. 2, No burnout vs. Medium-High levels; 1 vs. 3, No burnout vs. Profile 1; 1 vs. 4, No burnout vs. Profile 2; 2 vs. 3, Medium-High levels vs. Profile 1; 2 vs. 4, Medium-High levels vs. Profile 1; 3 vs. 4, Profile 1 vs. Profile 2*.

Then, the predictive capacity of demand variables and resource variables were analyzed on the 4 burnout levels (Table 2). In the first step, Sex, Age, and Level of education were included to control the size effect. On the other hand, the inclusion of demand variables (step 2) seemed to significantly improve the prediction of the model in all cases except Medium-High levels of burnout (NR^2^ = 0.01 to NR^2^ = 0.04) (which did not improve after the inclusion of any of the steps). In the second step, the inclusion of demands increased model prediction between 19% (Profile 1) and 26% (No burnout) in the other levels. *R*^2^ of Nagelkerke increased from 0.04 to 0.30 in No burnout levels, from 0.04 to 0.23 in Profile 1, and from 0.01 to 0.23 in Profile 2. The inclusion of resource variables increased the prediction of the model in No burnout, Medium-High level and Profile 1. The Nagelkerke *R*^2^ increased from 0.30 to 0.31 in No burnout level, from 0.04 to 0.05 in Medium-High level, and from 0.23 to 0.26 in Profile 1.

**Table 2:** Logistic regressions for no burnout, medium-high levels, profile 1, and profile 2.

**Variable**	**No burnout (Low levels burnout)**			**Medium-High levels (medium levels Burnout)**			**Profile 1 (Critical levels of burnout and no critical levels of guilt)**			**Profile 2 (Critical levels of burnout and critical levels of guilt)**		
	**B**	**SE**	**Wald**	**B**	**SE**	**Wald**	**B**	**SE**	**Wald**	**B**	**SE**	**Wald**
**Step 1**												
Sex	0.03	0.05	0.28	−0.04	0.05	0.71	−0.17	0.10	2.81	0.31	0.15	3.96*
Age	−0.02	0.003	56.57***	0.003	0.003	1.63	0.02	0.005	18.99***	0.02	0.008	3.75
L. of education	−0.37	0.03	117.10***	0.25	0.03	58.53***	0.72	0.08	88.78***	0.35	0.11	9.28**
*NR^2^*	0.04			0.01			0.04			0.01		
Cox & snell *R^2^*	0.03			0.009			0.02			0.003		
**Step 2**												
Sex	0.10	0.06	2.61	−0.06	0.05	1.36	−0.21	0.11	3.45	0.34	0.17	4.25*
Age	−0.02	0.003	50.38***	0.002	0.003	0.84	0.02	0.006	18.46***	0.02	0.009	4.26*
L. of education	−0.34	0.04	77.62***	0.23	0.03	44.07***	0.65	0.08	62.31***	0.20	0.12	2.78
EL	−0.17	0.05	12.88***	−0.003	0.04	0.006	0.21	0.09	5.01*	0.54	0.15	13.03***
IC	−0.77	0.07	131.46*	0.11	0.05	4.02*	0.25	0.10	6.16*	0.98	0.14	45.86***
Imbalance	−0.45	0.04	108.47***	0.21	0.04	30.40***	0.72	0.08	76.77***	−0.09	0.13	0.53
RC	−0.32	0.05	43.64***	−0.08	0.04	4.34*	0.38	0.08	25.99***	0.30	0.12	6.72**
RA	−0.73	0.05	221.65***	0.30	0.04	58.33***	0.59	0.07	70.13***	0.69	0.10	44.25***
Workload	−0.75	0.06	144.56***	0.22	0.05	16.62**	0.62	0.11	32.61***	0.94	0.17	30.93***
*NR^2^*	0.30			0.04			0.23			0.23		
Cox & snell *R^2^*	0.22			0.03			0.09			0.05		
**Step 3**												
Sex	0.08	0.06	1.57	−0.05	0.05	0.75	−0.13	0.11	1.33	0.35	0.17	4.39*
Age	−0.02	0.003	45.56***	0.002	0.003	0.50	0.02	0.006	17.66***	0.02	0.01	4.24*
L. of education	−0.35	0.04	79.16***	0.23	0.03	45.21***	0.68	0.08	66.51***	0.20	0.12	2.80
EL	−0.23	0.05	22.37***	0.02	0.04	0.33	0.38	0.09	15.71***	0.55	0.15	13.09***
IC	−0.72	0.07	110.27***	0.08	0.05	2.11	0.13	0.10	1.67	0.98	0.15	44.63***
Imbalance	−0.43	0.04	94.63***	0.19	0.04	24.88***	0.64	0.08	58.08***	−0.09	0.13	0.53
RC	−0.23	0.05	22.40***	−0.13	0.04	10.37***	0.17	0.08	4.52*	0.29	0.12	5.49*
RA	−0.52	0.05	96.61***	0.20	0.04	21.26***	0.23	0.08	8.39**	0.66	0.12	30.78***
Workload	−0.77	0.06	146.77***	0.22	0.05	16.35***	0.66	0.11	35.41***	0.93	0.17	29.93***
SS	0.12	0.04	10.04**	−0.05	0.03	2.33	−0.30	0.06	21.56***	0.04	0.10	0.15
Autonomy	0.52	0.06	80.48***	−0.21	0.05	18.90***	−0.68	0.09	52.88***	−0.13	0.15	0.79
Resources	0.06	0.04	2.22	−0.07	0.04	3.71	−0.21	0.08	6.99**	−0.02	0.12	0.03
*NR^2^*	0.31			0.05			0.26			0.23		
Cox & snell *R^2^*	0.23			0.03			0.10			0.05		

Freedom degree = 1. PE&I, Psychological exhaustion & Indolence; PE&G, Psychological exhaustion & Guilt; PE, Psychological exhaustion; L. of education, Level of education; EL, Emotional labor; IC, Interpersonal conflict; SS, Social support; RC, Role conflict; RA, Role ambiguity; B, regression coefficient; SE, Standard Error. NR^2^ = R^2^ of Nagelkerke.

* p ≤ 0.05;

** p ≤ 0.01;

*** *p ≤ 0.001*.

In the case of the results of No burnout level (Table 2), in the first step, the variables Age (*B* = −0.02) and Level of education (*B* = −0.37) were negative predictors of No burnout levels. In the second step, when demand variables were added to the analyses, the variables Age (*B* = −0.02), Level of education (*B* = −0.34), Emotional labor (*B* = −0.17), Interpersonal conflict (*B* = −0.77), Imbalance (*B* = −0.45), Role conflict (*B* = −0.32), Role ambiguity (*B* = −0.73), and Workload (*B* = −0.75) were predictors of No burnout levels. In the third step, the resource variables were added and the results showed that the demand variables Emotional labor (*B* = −0.23), Interpersonal conflict (*B* = −0.72), Imbalance (*B* = −0.43), Role conflict (*B* = −0.23), Role ambiguity (*B* = −0.52), and Workload (*B* = −0.77) predicted in a negative sense, while the resource variables Autonomy (*B* = 0.52) and Social support (*B* = 0.12) predicted in a positive sense belonging to that level of burnout. Likewise, the best predictors were Workload (*B* = −0.77) in a negative sense and Autonomy (*B* = 0.52) in a positive sense.

In the case of the results on Medium-High levels of burnout (Table 2), in the first step, Level of education (*B* = 0.25) was a predictor of Medium-High levels. In the second step, the variables Level of education (*B* = 0.23), Interpersonal conflict (*B* = 0.11), Imbalance (*B* = 0.21), Role conflict (*B* = −0.08), Role ambiguity (*B* = 0.30), and Workload (*B* = 0.22) were predictors of Medium-High levels of burnout. In the third step, resource variables were added to the analyses, and the results showed that the demand variables Imbalance (*B* = 0.19), Role ambiguity (*B* = 0.20), and Workload (*B* = 0.22) predicted in a positive sense, except Role conflict (*B* = −0.13) that predicted in a negative sense, while the resource variable Autonomy (*B* = −0.21) predicted in a negative sense the belonging to this level of burnout. Moreover, the best predictors were Workload (*B* = 0.22) and Role ambiguity (*B* = 0.20) in a positive sense and Autonomy (*B* = −0.21) in a negative sense.

On the other hand, with regard to the prediction for Profile 1 (Table 2), in the first step, the variables Age (*B* = 0.02) and Level of education (*B* = 0.72) were significant. Besides demand variables were added in the second step, the variables Age (*B* = 0.02), Level of education (*B* = 0.65), Emotional labor (*B* = 0.21), Interpersonal conflict (*B* = 0.25), Imbalance (*B* = 0.72), Role conflict (*B* = 0.38), Role ambiguity (*B* = 0.59), and Workload (*B* = 0.62) were predictors of this profile. In the third step, resources variables were added, and the results showed that the demand variables Emotional Labor (*B* = 0.38), Imbalance (*B* = 0.64), Role conflict (*B* = 0.17), Role ambiguity (*B* = 0.23), and Workload (*B* = 0.66) predicted in a positive sense, while the resources Social support (*B* = −0.30), Autonomy (*B* = −0.68) and Resources at work (*B* = −0.21) predicted in a negative sense the belonging to that level. Likewise, the best predictors were Workload (*B* = 0.66) and Imbalance (*B* = 0.64) in a positive sense and Autonomy (*B* = −0.68) in a negative sense.

In the case of Profile 2 (Table 2) the results showed that in the first step the variable Sex (*B* = 0.31) and Level of education (*B* = 0.35) were predictors of Profile 2. In the second step, when demand variables were added, the results showed that the variables Sex (*B* = 0.34), Age (*B* = 0.02), Emotional labor (*B* = 0.54), Interpersonal conflict (*B* = 0.98), Role conflict (*B* = 0.30), Role ambiguity (*B* = 0.69), and Workload (*B* = 0.94) were predictors of Profile 2. In the third step, when resource variables were added, the results showed that the demand variables Emotional labor (*B* = 0.55), Interpersonal conflict (*B* = 0.98), Role conflict (*B* = 0.29), Role ambiguity (*B* = 0.66), and Workload (*B* = 0.93) were positive predictors, while there were not any resources variables which acted as predictors. However, Sex (*B* = 0.35) and Age (*B* = 0.02) were positive predictors of this level of burnout. In addition, the best predictors were Interpersonal conflict (*B* = 0.98), and Workload (*B* = 0.93).

Finally, Sex, Age, Level of education, resources variables, and demand variables in the workplace that influenced the change from the lower levels of burnout (No burnout) to higher levels (Profile 2) were evaluated (Table 3). The results showed that all demand variables significantly influenced the moves between all burnout levels, with the exception of Emotional labor and Role conflict in the move from Profile 1 to Profile 2. In addition, the results were similar to resource variables. All results were significant, with the exception of the influence of Resources at work in the move from Profile 1 to Profile 2.

**Table 3:** Logistical regressions for the change of category.

**Variable**	**No burnout→Medium-High levels**			**Medium-High levels→Profile 1**			**Profile 1→Profile 2**		
	**B**	**SE**	**Wald**	**B**	**SE**	**Wald**	**B**	**SE**	**Wald**
**Step 1**									
Sex	−0.04	0.06	0.56	−0.12	0.11	1.21	0.46	0.18	6.27*
Age	0.01	0.003	24.88***	0.02	0.006	13.43***	−0.004	0.01	0.14
L. of education	0.37	0.04	103.18***	0.52	0.08	44.12***	−0.34	0.14	6.14*
*NR^2^*	0.03			0.03			0.02		
Cox & snell R^2^	0.02			0.02			0.01		
**Step 2**									
Sex	−0.10	0.06	2.52	−0.14	0.12	1.52	0.47	0.19	5.83*
Age	0.02	0.003	26.88***	0.03	0.006	18.36***	0.000	0.01	0.002
L. of education	0.36	0.04	77.55***	0.54	0.07	39.36***	−0.32	0.15	4.67*
EL	0.14	0.05	7.36**	0.25	0.10	6.72**	0.30	0.16	3.43
IC	0.65	0.07	83.38***	0.38	0.11	11.52***	0.69	0.16	17.55***
Imbalance	0.43	0.05	87.57***	0.63	0.09	49.60***	−0.61	0.15	17.71***
RC	0.22	0.05	19.18***	0.42	0.08	27.12***	0.01	0.13	0.01
RA	0.72	0.05	198.68***	0.52	0.08	45.86***	0.15	0.12	1.49
Workload	0.67	0.07	103.70***	0.65	0.12	30.87***	0.47	0.19	6.20*
NR^2^	0.24			0.22			0.12		
Cox & snell R^2^	0.18			0.12			0.08		
**Step 3**									
Sex	−0.08	0.07	1.35	−0.09	0.12	0.57	0.41	0.20	4.40*
Age	0.01	0.003	24.45***	0.03	0.006	16.46***	0.002	0.01	0.05
L. of education	0.37	0.04	78.69***	0.57	0.09	42.97***	−0.42	0.15	7.73**
EL	0.19	0.05	14.53****	0.42	0.10	16.95***	0.14	0.17	0.66
IC	0.59	0.07	66.32***	0.27	0.11	5.64**	0.81	0.17	22.17***
Imbalance	0.40	0.05	74.71***	0.55	0.09	38.12***	−0.61	0.15	16.39***
RC	0.13	0.05	6.45*	0.23	0.08	7.56**	0.22	0.14	2.40
RA	0.52	0.05	87.18***	0.22	0.09	6.48*	0.40	0.14	8.63**
Workload	0.70	0.07	107.91***	0.67	0.12	32.08***	0.47	0.19	5.30*
SS	−0.11	0.04	7.53**	−0.26	0.07	13.91***	0.35	0.12	8.01**
Autonomy	−0.52	0.06	71.46***	−0.56	0.10	31.88***	0.42	0.17	6.08*
Resources	−0.11	0.05	5.42*	−0.21	0.08	6.08*	0.19	0.14	1.85
NR^2^	0.26			0.25			0.16		
Cox & snell *R^2^*	0.19			0.14			0.11		

Freedom degree = 1. L. of education, Level of education; EL, Emotional labor; IC, Interpersonal conflict; SS, Social support; RC, Role conflict; RA, Role ambiguity; B, regression coefficient; SE, Standard Error. NR^2^ = R^2^ of Nagelkerke.

* p ≤ 0.05;

** p ≤ 0.01;

*** *p ≤ 0.001*.

The variable Workload seemed to be the most influential predictor in the moves from No burnout to Medium-High levels (*B* = 0.70) and Medium-High levels to Profile 1 (*B* = 0.67). However, the variable Interpersonal conflict seemed to be the most influential predictor in the change from Profile 1 to Profile 2 (*B* = 0.81) (Table 3, Step 3). All of the variables showed an influence on the movement between levels of burnout according to the hypothesized effect; however, 3 exceptions were found in the change from Profile 1 to Profile 2: (a) Imbalance showed a negative and significant effect (*B* = −0.61), (b) Social support showed a positive and significant effect (*B* = 0.35) and (c) Autonomy showed a positive and significant effect (*B* = 0.42).

## Discussion

Despite the statistics and cost of the different international and national institutes (European Agency for Safety Health at Work, 2016) that refer to a high loss in economy and society because of psychosocial risk, there is hardly any research about the burnout levels and the different psychosocial variables that can predict the progress of severity of burnout. Likewise, there are not a lot of studies about the influence of these psychosocial variables on the burnout process. These studies could help to understand better the process and create different interventions to reduce the cost and improve the employee's health and safety. Thus, the purpose of this study was to identify the demands and resource predictors of each burnout profile. On the other hand, it was to know the influence of the different demands and resource variables on the movements of one profile to another. The first aim of the study was performed through a cluster test, and the results showed that for the No burnout levels, each demand variable (Emotional labor, Interpersonal conflict, Imbalance, Role conflict, Role ambiguity, and Workload) were a negative sense predictor of this level, and resource variables (Social support and Autonomy) were a positive sense predictor of this type. In addition, variables Age and Level of education were a negative predictor of No burnout levels. In this case, the resource variables could be acting as protective variables, as other studies have shown (Setti et al., 2016; Khan et al., 2018). In addition, it shows the importance of having a healthy psychosocial environment at work, where the demands are controlled or are low, and resources are present.

Moreover, in the Medium-High burnout level, some demand variables (Imbalance, Role conflict, Role ambiguity, and Workload) and Autonomy were predictors. In particular, demand variables were a positive sense predictor, except Role conflict that was a negative predictor of Medium-High burnout level, and Autonomy was a negative sense predictor. Also, Level of education was a positive predictor of Medium-High burnout level.

Likewise, some demand variables (Emotional labor, Imbalance, Role conflict, Role ambiguity, and Workload) and resource variables (Social support, Autonomy, and Resources at work) predict Profile 1, specifically, demand variables except in a positive sense and resource variables in a negative sense. The results showed that the variable Age is a predictor of Profile 1; to be older seems to predict burnout.

For Profile 2, some of the demand variables (Emotional labor, Interpersonal conflict, Role conflict, Role ambiguity, and Workload) were found as a positive predictor. In addition, the results showed that the variables Sex and Age are a predictor of Profile 2. In particular, being a woman seems to predict burnout. These results are similar to other researches (Alavinia and Ahmadzadeh, 2012; Leineweber et al., 2013; Nava and Páez, 2013).

Therefore, Hypothesis 1 and Hypothesis 2 were accepted. These results agree with some of the results of studies about the relationship between demand variables (Skaalvik and Skaalvik, 2007; Backhaus et al., 2018; Vullinghs et al., 2018) and resource variables (Khan et al., 2018) and burnout. They showed the importance of the demand variables in burnout prediction (Demerouti et al., 2001), increasing the probability of having the highest levels of burnout when there are high levels of demands (Emotional labor, Interpersonal conflict, Imbalance, Role conflict, Role ambiguity, and Workload). Furthermore, these results showed that the most important demand variable that predicts burnout in almost all cases was Workload. Thus, Workload should be controlled to reduce the probability to develop burnout.

The second aim of this study was to identify the influence of the predictors in the moves between levels of burnout. In the movements between levels of burnout, results showed that the demand variables were present in each movement. Moreover, the demand variable Interpersonal conflict showed the highest influence in the movement from Profile 1 to Profile 2, and the Workload variable showed the highest influence in the movements from No burnout to Medium-High level and from Medium-High level to Profile 1. All resource variables influenced all the movements between profiles, with the exception of Resources at work in the movement from Profile 1 to Profile 2. Therefore, the demand and resource variables were predictors of a change in the level of burnout. Workload was present in all cases. So, Hypothesis 3 is confirmed. However, the variable Imbalance has a negative influence in the change -i.e., lower levels of imbalance increasing levels of critical burnout. On the other hand, in the move from Profile 1 to Profile 2, the resource variables Social support and Autonomy were positively significant.

Furthermore, low levels of imbalance in conjunction with high levels of social support and autonomy influence the move from Profile 1 to Profile 2. This result can be explained because teachers with higher levels of burnout, when they perceive low levels of imbalance (e.g., *I receive a lot of compensation for the care and attention I give to my students* or *I am rewarded a lot of for the effort I make at work*), high levels of social support and high levels of autonomy might be attributing to themselves all the responsibility about their negative behavior toward the students. Because the organizational environment is supportive, their feelings of guilt rise up, increasing their burnout levels as well. This result gives a new view of the burnout process to understand the critical burnout levels, showing that feelings of guilt have an influence on the increment of the unhealthier burnout levels. These results will help to prevent and create intervention programs because Social support and Autonomy should not be used in the Profile 2 intervention. Some studies showed similar results in nurses where the Social support did not prevent burnout when there are high levels of Workload (Fong, 2016).

These results showed the importance of some resource variables as a protector variable on the no chronic burnout, and they could help to design an intervention plan to stop a person from developing a chronic burnout syndrome (e.g., Gil-Monte, 2019b). They also help to understand how burnout is changing from one statement to another.

This study could be helpful in understanding the variables that actually affect the different burnout levels, and it can be used to create a specific intervention for each profile. In addition, it shows which variables are important to reduce and prevent burnout syndrome. Social support should be used to prevent burnout, but it should not be used for chronic burnout intervention because this study showed that it would not help to improve the higher levels of burnout. The intervention for chronic burnout should focus on reducing some demand variables such as Workload and Interpersonal conflicts, but also reducing some resource variables such as Social support and Autonomy in the highest or critical levels of burnout. However, the intervention in low levels of burnout should focus on increasing some resource levels to prevent chronic levels of burnout.

Results from this study could help to design intervention programs that promote the health of the teachers. In addition, the prevention of burnout syndrome would help to reduce the social and organizational costs. Also, this study could help to understand how burnout syndrome develops and help future studies to know the important variables that influence burnout syndrome.

Our results provide useful data in terms of their practical implications since, on the one hand, they allow to identify the key elements of the burnout development process at different stages, highlighting those in which it would be convenient to intervene in order to prevent the onset of burnout (such as social support) or to prevent its worsening (such as autonomy), as well as important elements throughout the whole process (such as workload and interpersonal conflicts). We have also seen the importance of the element of guilt and how some factors are related to its development (such as social support). The present study allows to discern the key intervention factors depending on the characteristics of each particular case: if we want to prevent the development of burnout, if burnout already exists and we want to reduce its levels or prevent its worsening, if guilt is present or not, among others. The design of strategies adapted to the different cases allows these to be more efficient, optimizing the use of the resources that such strategies require.

This study is not without limitations. Firstly, a non-probabilistic method in a single region of Spain was used to get the sample, so it is difficult to generalize these results. Secondly, the results of this study were obtained with self-report. Therefore, it can produce some bias in the results. Thirdly, the variable Sex could contaminate the results as 72.5% of the sample were women. Fourthly, the sample was collected for a long period of time that could negatively influence the results. Finally, the absence of longitudinal data does not allow causal inferences to be made; therefore, the predictions observed in our analyses are predictions of the statistical variance observed in the scores, which allows a better understanding of the role of some variables in the development or worsening of burnout, but they do not allow for a cause-and-effect interpretation. Future studies should consider these limitations and extend the sampling to other geographical and cultural contexts to replicate this study so it can be helpful to other professional areas. Likewise, it would be interesting to have other types of objective measures or those coming from external observers. Despite all this, the study is of special interest considering the large size of the sample under study, as well as the results obtained on the role that sociodemographic variables, demands, and resources have in the appearance of burnout, and something more novel, to know those variables that seem to be involved in the evolution toward more harmful profiles of burnout.

## Data Availability Statement

The raw data supporting the conclusions of this article will be made available by the authors, without undue reservation.

## Ethics Statement

This study respected the fundament principles of the Declaration of Helsinki (World Medical Association, 2013), with particular emphasis on the anonymization of the data collected, confidentiality and non-discrimination of participants. All the educational non-university centers in the Community of Valencia were asked to participate and the teacher's collaboration was voluntary. Teachers were informed about the purpose of the study, the possible benefits and possible consequences of their participation. This study was part of a psychosocial assessment of the Instituto Valenciano de Seguridad y Salud en el trabajo (INVASSAT). In all cases regional government instructions were followed. Previously to start the assessment, the ethical department of this institution was consulted and concluded that as the participation was voluntary and the teachers just needed to answer an anonym questionnaire, the research did not need to be checked by a bioethics committee.

## Author Contributions

AS-R and ML-P wrote the article and prepared to submit. PG-M got the sample and helped to write the article. All authors contributed to the article and approved the submitted version.

## Conflict of Interest

The authors declare that the research was conducted in the absence of any commercial or financial relationships that could be construed as a potential conflict of interest.
